# In Search of Clinical Impact: Advanced Monitoring Technologies in Daily Heart Failure Care

**DOI:** 10.3390/jcm10204692

**Published:** 2021-10-13

**Authors:** Dean Nachman, Eldad Rahamim, Yotam Kolben, Bethlehem Mengesha, Gabby Elbaz-Greener, Offer Amir, Rabea Asleh

**Affiliations:** 1 Hadassah Medical Center, Faculty of Medicine, Heart Institute, Hebrew University of Jerusalem, Jerusalem 91120, Israel; eldad.rahamim@gmail.com (E.R.); bettytade@gmail.com (B.M.); gabby@hadassah.org.il (G.E.-G.); oamir@hadassah.org.il (O.A.); 2 Hadassah Medical Center, Department of Medicine, Faculty of Medicine, Hebrew University of Jerusalem, Jerusalem 91120, Israel; yotamkol@hadassah.org.il; 3 Azrieli Faculty of Medicine, Bar-Ilan University, Ramat-Gan 5290002, Israel

**Keywords:** heart failure, remote patient monitoring, telemedicine, preventive medicine, rehospitalization, novel technologies

## Abstract

Despite significant advances in the management of heart failure (HF), further improvement in the outcome of this chronic and progressive disease is still considered a major unmet need. Recurrent hospitalizations due to decompensated HF frequently occur, resulting in increased morbidity and mortality rates. Past attempts at early detection of clinical deterioration were mainly based on monitoring of signs and symptoms of HF exacerbation, which have mostly given disappointing results. Extensive research of the pathophysiology of HF decompensation has indicated that hemodynamic alterations start days prior to clinical manifestation. Novel technologies aim to monitor these minute hemodynamic changes, allowing time for therapeutic interventions to prevent hemodynamic derangement and HF exacerbation. The latest noticeable advancements include assessment of lung fluid volume, wearable devices with integrated sensors, and microelectromechanical systems-based implantable devices for continuous measurement of cardiac filling pressures. This manuscript will review the rationale for monitoring HF patients and discuss previous and ongoing attempts to develop clinically meaningful monitoring devices to improve daily HF health care, with particular emphasis on the recent advances and clinical trials relevant to this evolving field.

## 1. Introduction

Heart failure (HF) is a common clinical syndrome with detrimental effects at the individual patient and society levels [1,2]. Overall, 2.2% of US adults or nearly 6.2 million individuals suffer from HF, imposing a significant yearly financial burden estimated at 30.7 billion US dollars in 2012, and projected to more than double by 2030 [3]. The prevalence of HF is expected to continuously grow due to medical and societal developments [3]. First, HF is rising as the population ages, reaching more than 12% in older adults above the age of 80 [4]. Second, the improvement in treatments of HF has led to lower mortality rates, leaving more patients in need of chronic care [5]. Third, the rise in obesity and metabolic syndrome incidence is another contributor to the increased prevalence of HF cases [3,6,7,8]. Recently, advances in HF drug and device therapies have brought about impactful achievements to the field, yet a quarter of patients will endure considerable symptoms, hospitalizations, and mortality, despite optimal medical treatment. Consequently, additional approaches to further improve the management of HF are essential [6,9,10].

HF is characterized by a progressive course of disease accompanied by recurrent exacerbations leading to high hospitalization and rehospitalization rates, which account for a substantial part of the disease load [11,12,13,14,15]. Among the elderly, acute decompensated heart failure (ADHF) is the leading cause of hospitalization [3]. The 30-day rehospitalization rate following the first admission to the hospital for HF exacerbation is 22–29.4%, which is the most common amongst all other etiologies [16,17,18,19]. Likewise, ADHF is a leading cause (8.6%) of rehospitalization following hospitalization for other etiologies [16]. Moreover, ADHF admission is associated with poor quality of life, and approximately one-third of patients die within a year after an index admission [20,21,22]. Given the high prevalence and financial incentive, reducing HF hospitalization and readmission rates has become a foremost priority in the health systems [23,24].

**Figure 1:**
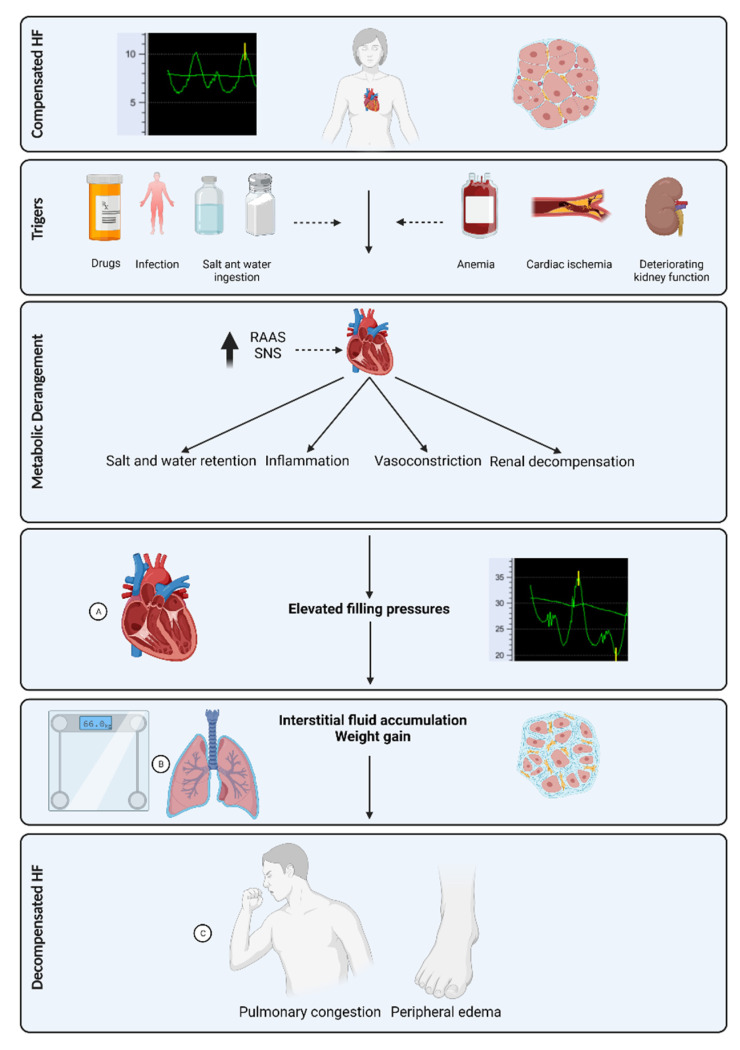
Pathophysiologic cascade of heart failure decompensation. Normal cardiac filling pressures and interstitial fluid is noted when heart failure is compensated. Elevated cardiac filling pressures and interstitial fluid can be noticed prior to symptomatic compensated heart failure. (**A**): Elevated cardiac filling pressures can be monitored using implantable pressure sensors. (**B**): Interstitial fluid accumulation can be monitored using bioimpedance or remote dielectric sensing and electronic internet connected scales can be used for monitoring weight. (**C**): Symptoms can be monitored when overt decompensated heart failure ensues. RAAS: renin angiotensin aldosterone system; SNS: sympathetic nervous system. HF: heart failure.

The pathophysiological process of ADHF incites days to weeks prior to the development of noticeable signs and symptoms [25,26,27]. In a simplified approach (Figure 1), a precipitating factor, such as ischemia, acute renal failure, increased salt consumption, worsening anemia, or infection, may result in cardiac function deterioration and augmentation of compensatory mechanisms. Both rennin-angiotensin-aldosterone pathway and sympathetic nervous system activation induce salt and water retention, vasoconstriction, inflammation, and renal decompensation [28,29]. In the course of time, fluid retention elevates cardiac filling pressures, which backflow to the lungs and the systemic venous system, ultimately leading to interstitial edema, weight gain, and eventually to pulmonary edema [23,30].

Understanding the time course of progression to ADHF, it has been hypothesized that interventions to achieve an euvolemic status prior to overt clinical manifestation may prevent HF exacerbation events [31,32]. Several approaches to monitor HF patients have been tested, aiming to detect early warning signs of HF exacerbation. Daily monitoring of weight gain has not been successful in reducing rehospitalization or death rates as compared to control cases in weight monitoring in patients with severe heart failure (WISH) trial involving 344 in-hospital patients with ADHF [33]. In other large-scale studies, weight monitoring did not predict HF rehospitalizations [34].

In the Trans-European Network-Home-Care Management System trial (TEN-HMS), 426 patients with recent HF-related hospitalizations were randomized into three monitoring options: home telemonitoring (HTM), monthly nurse telephone support (NTS), or usual care (UC). Home telemonitoring (consisting of twice-daily measurements of body weight, blood pressure, and heart rate and rhythm by automated devices) was reviewed by a care manager at a linked medical reference center to facilitate prompt intervention when needed. Patients randomized to HTM or NTS had significantly lower mortality rates at 240 days post hospitalization compared with UC (29%, 27%, and 45%, respectively, *p* = 0.032). There was no difference in mortality or HF admission between HTM and NTS, but the length of stay in the hospital was six days shorter for the HTM arm [35]. Supporting results were also obtained from the Telemedical Interventional Management in Heart Failure II (TIM-HF2) trial, and in trials focusing on the Heart-Mobile program, demonstrating improved hospitalization indices and reduced all-cause mortality in similarly monitored participants [36,37]. In contrast, the Better Effectiveness After Transition–Heart Failure (BEAT-HF) randomized trial involving 1437 patients who were discharged home with comparable monitoring methods after HF hospitalization has failed to demonstrate a favorable clinical outcome at 180 days of follow-up [38].

Because many conservative monitoring modalities focused on detecting outputs late in the pathophysiological cascade of ADHF have shown conflicting results, it has been suggested that focusing on physiological signals related to earlier alterations may give better results [26]. Therefore, this article aims to review the most recent advances and future perspectives regarding this evolving field of HF monitoring.

## 2. Non-Invasive HF Monitoring

### 2.1. Lung Fluid Volume Assessment

The fluid content range in the lungs is 20 to 35% in normal conditions, above which pulmonary edema may occur [23]. Residual pulmonary congestion at the time of discharge after hospitalization for ADHF is a strong predictor of rehospitalization [39]. Pulmonary congestion develops prior to clinical evidence of ADHF (Figure 1), thus making it an attractive target for monitoring by several non-invasive technologies.

Impedance techniques in lung water measurements are based on the principle that air and water have different resistance. When water fills the lungs, conductance increases and impedance decreases [40,41,42,43]. Lung impedance (LI) monitoring using Edema-Guard monitor (CardioSet Company Ltd., Tel Aviv, Israel) once a month at ambulatory clinic visits demonstrated decreased HF hospitalization and mortality rate in a randomized controlled trial involving 256 HF patients [44]. Moreover, as measured by LI during HF hospitalization, the improvement in pulmonary fluid volume was predictive of lower readmission rate and demonstrated a better correlation than other clinical measures, such as N-terminal pro-brain natriuretic peptide (NT pro-BNP) or weight [45,46,47].

The CoVa™ Monitoring System (toSense™, Inc. San Diego, CA, USA) is a wearable necklace-shaped device that monitors electrocardiography (ECG) and calculates stroke volume, cardiac output, and thoracic fluid index (TFI) using chest bioimpedance. In a pilot study to predict HF events, 20 patients with New York Heart Association class I-IV HF symptoms underwent daily home monitoring with The CoVa™ Monitoring System. The TFI volatility, defined as the standard deviation of the TFI determined over five days divided by the average TFI over the same 5-day period, was recorded. This study showed an increase of ≥40% in TFI volatility before 100% of the HF events and an 8% reduction in stroke volume before 60% of ADHF events. These findings suggest that a multisensory system has the potential to predict HF hospitalizations [48].

**Figure 2:**
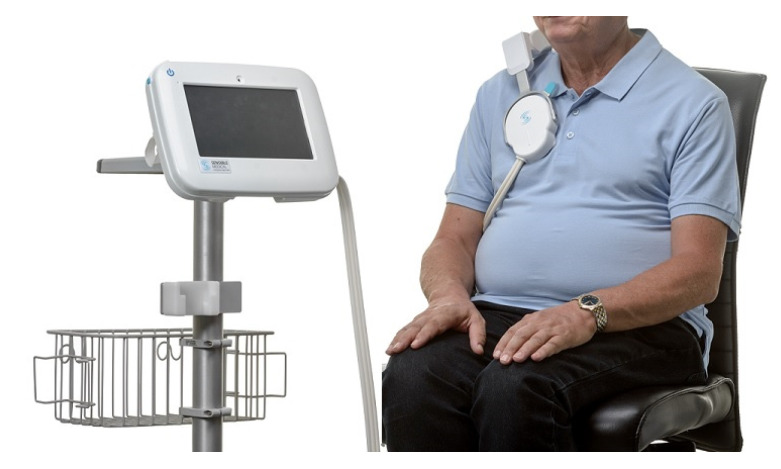
The Remote Dielectric Sensing (ReDS) vest (Sensible Medical Innovations Ltd., Netanya, Israel) assesses lung fluid content using electromagnetic-based technology. Reproduced with permission from Sensible Medical Innovations Ltd. 2021, all rights reserved.

The Remote Dielectric Sensing (ReDS) vest (Sensible Medical Innovations Ltd., Netanya, Israel), measures the lung fluid content using a focused electromagnetic beam similar to radar technology (Figure 2). When compared to right heart catheterization in HF patients, readings of >34% fluid content were highly correlated to pulmonary capillary wedge pressure >18 mmHg (area under the curve (AUC) of 0.848, a sensitivity of 90.7%, and a specificity of 77.1%) [49]. Furthermore, results from 24 patients hospitalized for ADHF showed a correlation between the reduction in ReDS values and reduced pulmonary congestions and net fluid balance [50]. In another study, including 47 patients hospitalized for HF, hospitalization rates before and after the index hospitalization were compared without and with the use of ReDS vest, showing a significant reduction in HF readmissions with ReDS vest technology [51]. Additionally, preliminary results from a multicenter trial randomizing 268 patients to monitoring-guided or standard medical therapy following ADHF hospitalization demonstrated a 48% (95% CI: 31–87%, *p* = 0.01) reduction in 9 months rehospitalization in the ambulatory ReDS monitoring group [52].

### 2.2. Whole-Body Electrical Bioimpedance

Whole-body bioimpedance (WBBI) allows the non-invasive measurement of body composition for the assessment of various clinical conditions. This technology works by sending a small electrical current throughout the entire body and measuring its impedance. WBBI was first introduced by Hoofer then revised by Nyboer and validated by Lukaski [53,54,55,56,57] before it was utilized in cardiology [58,59,60,61]. The NICaS monitor (NI Medical; Hod-Hasharon, Israel) is a continuous WBBI system characterized by the capability of measuring impedance fluctuations through two electrode-like sensors applied to the lower and upper limbs simultaneously with a three-lead ECG [62]. The system provides real-time data on the patient’s cardiovascular function, including heart rate, stroke volume and stroke index, cardiac output, and cardiac index. The system also provides information regarding total peripheral resistance, total body water, and the Granov Goor index, an indicator of left ventricular (LV) function [63]. Several studies comparing cardiac output measurements using the NICaS system with the traditional thermodilution method found a correlation of r = 0.886 − 0.91 and Bland-Altman limits of agreement of −1.06 and 0.68 L/min [62,64,65]. The system also detected cardiac index changes during coronary artery bypass grafting operations and has monitored the hemodynamic effects of vasodilator administration in patients suffering from ADHF [62]. In an additional study, the device managed to reliably monitor 46 patients hospitalized for ADHF during an infusion of a novel second-generation nitroxyl donor with inotropic, lusitropic, and vasodilatory effects [66]. Contradicting data come from a study comparing the performance of this device to cardiac magnetic resonance imaging (MRI), showing only moderate correlation to cardiac function with significant variability among patients [67].

### 2.3. Piezoelectric Sensor for Physiologic Vibration Monitoring

This sensor uses the piezoelectric effect and converts changes in pressure into electrical signals [68]. Vibration is generated inside the chest wall by the heart and lungs, pumping blood and air. EverOn (EarlySense, Ramat Gan, Israel) is a novel under-the-mattress piezoelectric sensor that can sense these subtle physiological vibrations and convert them to an electrical signal ready to be decoded at a control unit [69]. In a single-center study involving 30 HF patients discharged home following hospitalization for HF exacerbation, 640 nights of monitoring were collected and analyzed. The study found patterns that could be unique among patients at risk for readmission due to HF exacerbation. For example, respiratory rate was a significant risk factor for HF readmission [70].

### 2.4. Wearable Devices

We increasingly observe inflation in complex wearable monitoring devices [71]. Numerous consumer smartwatches incorporate medical-grade sensors such as ECG and pulse oximeters, enabling early detection of common medical conditions such as atrial fibrillation [72,73,74]. ECG patches allow long-term ambulatory monitoring of arrhythmia [75]. In addition, continuous blood pressure monitoring is available at a wristwatch configuration using cuff-based or pulse wave transit time technologies [76,77].

The LINK-HF (Multisensor Non-invasive Remote Monitoring for Prediction of Heart Failure Exacerbation) multicenter study has examined the accuracy of a wearable multisensor remote monitoring patch (Vital Connect, San Jose, CA, USA) for the prediction of ADHF rehospitalization. This disposable patch is attached to the chest and is able to collect continuous ECG, skin impedance, body temperature, and activity level data. Utilizing artificial intelligence to generate a personalized baseline model-based alerts, the system forecasted ADHF with a 76% to 88% sensitivity and an 85% specificity [78]. This study emphasizes the power of personalized data collection and analysis as well as the clinical usefulness of a multiparameter approach that has become available with the introduction of these wearable devices in clinical practice.

## 3. Invasive HF Monitoring

### 3.1. Cardiac Implanted Electronic Devices

Cardiac implanted electronic devices (CIEDs) are indicated in numerous HF patients. Alongside the competence to monitor rhythm disturbances and sleep-disordered breathing, several studies have examined whether CIED’s remote monitoring capabilities could facilitate early warning of ADHF development. In the IN-TIME trial (implant-based multiparameter telemonitoring of patients with heart failure), HF treatment was modified according to home-monitored variations in the electrocardiogram, such as ventricular extrasystoles or tachyarrhythmias, and patient activity level. At one year of follow-up, 63 (18.9%) of 333 monitored patients vs. 90 (27.2%) of 331 in the control group developed the composite outcome of death, HF hospitalization, worsening NYHA class, or worsening self-assessment (OR 0.63, 95% CI: 0.43–0.90, *p* = 0.013) [79]. However, other studies utilizing CIEDs with similar capabilities have failed or showed mixed results [80].

A unique feature of selected CIEDs is the capability to perform intrathoracic impedance monitoring, to some extent, similar to the non-invasive devices already described [81,82]. In the Diagnostic Outcome Trial in Heart Failure (DOT-HF), 335 HF patients implanted with CIEDs capable of impedance monitoring (OptiVol fluid assessment algorithm, Medtronic Inc., Minneapolis, MN, USA) were randomized to active monitoring or no monitoring. Unexpectedly, active monitoring was found to be associated with higher rates of HF hospitalization and without a mortality benefit [83]. It has been proposed that these findings might be explained by having an extremely high sensitivity with low specificity of these CIEDs, leading to overdiagnosis of worsening HF and increased rates of hospitalization for HF [84,85]. Furthermore, it was suggested that monitoring the complex interplay of HF with a single signal is not sufficient. To overcome these limitations, the HeartLogic algorithm (Boston Scientific, Marlborough, MA, USA) combines multiple pathophysiology-driven parameters into an integrated index, including heart sounds, respiration and heart rate, thoracic impedance, and physical activity. The HeartLogic algorithm was able to detect ADHF events with a sensitivity of 70% with a median lead time of 34 days prior to the event, and small reports also demonstrated improved clinical outcome when HF management was guided by the algorithm [86,87]. 

### 3.2. Pulmonary Artery Pressure Monitoring

Notably, previous studies have demonstrated that an increase in filling pressure may precede HF decompensation by three weeks or even more [27]. These findings have accelerated the search for effective and reliable devices that can consistently transmit intracardiac pressure readings to identify filling pressure rise early before clinical deterioration occurs. Two decades ago, an implantable hemodynamic monitoring device implanted in the right ventricle as a pacemaker was introduced. The device recorded various parameters, such as heart rate, right ventricle systolic and diastolic blood pressure, and estimated diastolic pulmonary artery (PA) pressure [88,89]. This device opened the door for an era of advanced invasive monitoring in the setting of HF.

**Figure 3:**
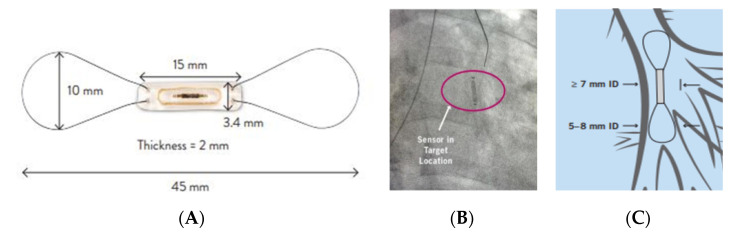
The CardioMEMS HF System. (**A**) The CardioMEMS HF System (Abbott, Sylmar, CA, USA) for pulmonary artery pressure monitoring; fluoroscopy imaging (**B**); and illustration (**C**) of the device situated to the left descending pulmonary artery. HF: heart failure. Source: CardioMEMS is a trademark of Abbott or its related companies. Reproduced with permission from Abbott, 2021. All rights reserved.

The CardioMEMS (Abbott, Sylmar, CA, USA) (Figure 3) is a wireless pressure sensor that uses micro-electromechanical systems (MEMS) technology. The device is implanted in the distal PA via a right heart catheterization. It is composed of a coil and a capacitor enveloped by a sealed silica capsule covered by silicone. Changes in PA pressure are detected by the sensor and transmitted to a home-monitoring device. The data are collected by the patient and transferred to the clinic for prompt analysis and adjustment of HF therapies accordingly [90].

The CardioMEMS HF Sensor Allows Monitoring of Pressures to Improve Outcomes in NYHA Functional Class III Heart Failure Patients (CHAMPION) trial has investigated the use of CardioMEMS sensor implanted in the PA. Five hundred and fifty HF patients with NYHA Class III and a previous admission for HF in the prior year were recruited. On top of standard care and after implementing the CardioMEMS sensor in both groups, in the treatment group, physicians could monitor the PA pressure and adjust treatment accordingly, while in the control group, no PA pressure-guided adjustment of treatment was performed. After six months of follow-up, there was a significant reduction in hospitalization for HF in the treatment group (HR 0.72, 95% CI 0.60–0.85, *p* = 0.0002) compared to the control group. No major complications related to the sensor implantation or any sensing failure were observed. During the entire follow-up period (mean of 15 months), the treatment group had a 37% reduction in HF-related hospitalizations compared with standard care alone [91]. In the open-access period, which followed patients for an additional 13 months, physicians had access to PA pressures for all patients. Patients who were not previously monitored while in the control group had a 48% (HR 0.52 (95% CI: 40–69%; *p* < 0.0001) reduction in admissions for HF compared with the nonmonitored period [92]. These results were consistent in all patients regardless of the LV ejection fraction or of receiving guideline-directed medical therapy [93,94].

To assess the generalizability of the CardioMEMS system, a larger study involving 1200 participants was conducted, with a special focus on subgroup diversity defined by sex, race, and ejection fraction. The rate of HF-related hospitalizations was significantly lower after 12 months of follow-up compared with the year before implantation (0.54 vs. 1.25 events per patient-years, hazard ratio (HR) 0.43 (95% CI: 0.39–0.47; *p* < 0.0001)). The rate of all-cause hospitalization was also lower following sensor implantation (1.67 vs. 2.28 events per patient-years, HR 0.73 (95% CI: 0.68–0.78), *p* < 0.0001)). Subgroup analyses including ejection fraction, sex, race, cause of cardiomyopathy, presence/absence of implantable cardiac defibrillator, or cardiac resynchronization therapy found no significant interaction between any of these variables and the prespecified outcomes, indicating consistent benefits on HF hospitalization using the CardioMEMS system in various patient cohorts [95].

Given these promising results, CardioMEMS was approved by the FDA in 2014 [96], and real-world data from several retrospective cohort studies demonstrated similar results [97,98]. In the European Society of Cardiology (ESC) guidelines from 2021, monitoring of PA pressure using a wireless implantable hemodynamic monitoring system (CardioMEMS) received a class IIb recommendation for symptomatic patients with HF and a previous HF hospitalization [99].

The recent 1022 participants, GUIDE-HF (hemodynamic-guided management of HF) trial tested if the benefits of PA monitoring extended beyond NYHA functional class III to patients with NYHA Functional class II and IV. The primary endpoint of all-cause mortality and HF hospitalization or unplanned hospital visit was not met in the hemodynamic-guided management compared to controls (HR 0·88, 95% CI 0·74–1·05; *p* = 0·16). Importantly, the trial was conducted during the COVID-19 pandemic and prespecified COVID-19 impact analysis found significant improvement in the treatment group prior to the pandemic (HR 0·81, 95% CI 0·66–1·00; *p* = 0·049) [100].

The Cordella System is another wireless PA pressure sensor. In one study, the Cordella device was implanted in 30 patients without complications or sensor failure. At 90 days, there was a mean difference of 2.7 mmHg between the Cordella sensor and Swan-Gantz catheter measurements (Cordella Sensor: 22.5 ± 11.8 mmHg; Swan–Ganz catheter: 25.2 ± 8.5 mmHg) [101].

### 3.3. Left Atrial Pressure Monitoring

Whereas CardioMEMS and other PA sensors measure right-sided pressures, left-side filling pressure measurements may provide additional important information regarding the patient’s tendency for pulmonary congestion. In animal models, an increase in left atrial (LA) pressure significantly correlated with pulmonary congestion, and reversal of pressure elevation resulted in normalization of lung permeability. In addition, many factors contribute to a mismatch between PA and LA pressure, including elevated pulmonary vascular resistance, advanced HF, acute HF, and pulmonary hypertension [26]. These data suggest that PA pressure measurements may be inaccurate in estimating LV filling pressure.

The HeartPOD (Savacor Inc., Los Angeles, CA, USA) is a system that allowed direct LA pressure using an implantable sensor lead connected to a subcutaneous module. The sensor was implanted in the LA transvenously through the interatrial septum [102]. In a large randomized controlled trial examining the safety and efficacy of the HeartPOD system in patients with NYHA class III HF symptoms, patients’ enrollment stopped early due to excess implant-related complications [103]. Preliminary results failed to show a net clinical benefit using this device. However, positive results were noted when retrospectively analyzing the CHAMPION trial endpoints of HF-related hospitalizations, thus leaving hope for LA pressure measurement as an optional tool for effective HF monitoring [104].

**Figure 4:**
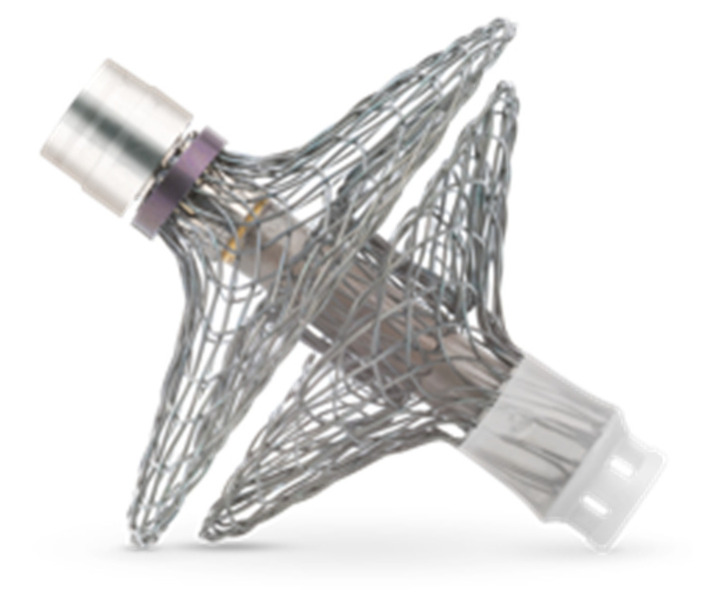
The V-LAP (Vectorious Medical Technologies, Tel Aviv, Israel) wireless left atrial pressure sensor is meant for implantation to the inter-atrial septum. Reproduced with permission from Vectorious Medical Technologies, 2021. All rights reserved.

A novel LA pressure sensor that is currently being investigated is the V-LAP system (Vectorious Medical Technologies, Tel Aviv, Israel). The V-LAP system is a wireless sensor that uses a MEMS pressure transducer and is implanted in the interatrial septum under angiographic and echocardiographic guidance (Figure 4). In preclinical phases, the V-LAP system was implanted in 10 ovines, and its measurements were compared with postcapillary wedge pressure (PCWP) obtained by right heart catheterization at 1, 2, and 3–6 months after implantation. The mean difference was 0.19 ± 2.51 mmHg, and a strong correlation between V-LAP and PCWP measurements was observed, with r = 0.97 [105]. Short reports regarding patients implanted with the V-LAP system have similarly indicated significant correlations between PCWP measurements using right heart catheterization and LA pressure measurements, and when appropriate, clinical responses to an increased dose of diuretics in patients with high pressures measured by V-LAP has been observed [106,107]. An ongoing single-arm, open-label pilot clinical trial, the VECTOR-HF (V-LAP Left Atrium Monitoring systEm for Patients With Chronic sysTOlic & Diastolic Congestive heaRt Failure), is designed to assess the safety of the V-LAP system in patients with HF.

## 4. Conclusions

Early detection and intervention in HF patients to prevent clinical HF decompensation and subsequent hospitalization may provide significant health and financial advantages. In contrast to previous monitoring methods, novel technologies have been developed to target the initial aspects of the pathophysiological cascade of HF decompensation. Invasive and non-invasive methods have remarkably advanced cardiovascular medicine, taking advantage of recent developments in MEMS, big data, artificial intelligence, and wearable sensors. There is a growing body of evidence supporting a potential clinical benefit from monitoring devices for the management of HF, mainly with PA pressure monitoring. Table 1 summarizes the key clinical trials published recently on different ambulatory heart failure monitoring technologies and their main findings. In the future, alongside further technological advances, appropriate integration of patient monitoring into the clinical workflow will help make the most of these exciting devices.

## Figures and Tables

**Table 1:** Key recent clinical trials on ambulatory heart failure monitoring technologies.

Year	Reference	Patient Characteristics	Monitoring Method	Follow Up	Primary Endpoint	Secondary Endpoint
2012	WISH [33]	344 patients hospitalized for ADHF and NFYHA III-IV, LVEF < 50%	Daily weighing using internet connected scale.	12 months	No difference in cardiac re-hospitalizations (HR 0.90, CI 0.65–1.26, *p* = 0.54)	No difference in all cause hospitalization, death, or composite of both.
2005	TEN-HMS [35]	426 patients with in 6 weeks of ADHF admission and LVEF <40% and on diuretics	Home telemonitoring (automatic BP, electronic scale, ECG), monthly nurse phone call or usual care	240 days	Days lost for death or hospitalization did not differ (12.7%, 15.9%, 19.5% respectively)	Mortality was higher in usual care group (45 vs. 27% in nurse phone call and 29% in telemonitoring groups)
2016	BEAT-HF [38]	1437 patients hospitalized for ADHF	electronic telemonitoring (BP, heart rate, weight, symptoms) + monthly tele-coaching or usual care	180 days	Similar all cause hospitalization at 180 days- 50.8% vs. 49.2% respectively (HR-1.03; 95% CI, 0.88–1.20; *p* = 0.74)	no significant differences in 30-day readmission or 180-day mortality.
2016	IMPEDANCE-HF [44]	256 patients with ADHF admission in the last year, LVEF < 35%, NYHA II-IV	Monthly lung impedance vs. usual care	48 ± 32 months	211 vs. 386 ADHF hospitalizations (*p* < 0.001) among monitored vs. control	42 vs. 59 deaths respectively (HR 0.52, 95% CI 0.35–0.78, *p* = 0.002)
2019	SMILE [52] (Preliminary results)	268 patients with current ADHF hospitalization	Remote dielectric sensing vs. usual care	6.1 ± 3.4 months	21 vs. 43 readmissions (HR 0.52, 95% CI- 0.31–0.87, *p* = 0.01)	No mortality benefits. Lower days lost for ADHF (1.37 vs. 2.62, *p* = 0.006)
2014	IN-TIME [79]	664 patients, LVEF < 35%, NYHA II-III, OMT.	CIED based daily monitoring (HR, activity, arrythmia, HR, HR variability, HR at rest, ventricular ectopy) vs. usual care	12 months	Composite of all-cause death, overnight hospital admission for heart failure, change in NYHA class patient global self-assessment was better in monitored group (18.9% vs. 27.2%, OR 0.63, 95% CI 0.43–0.90, *p* = 0·013)	Mortality of 10 vs. 27 patients respectively.
2011	DOT-HF [83]	335 patients with ADHF admission in the last year, LVEF < 35%, NYHA II-IV	CIED based thoracic impedance monitoring vs. usual care	14.9 ± 5 months	all-cause mortality and HF hospitalizations was similar (29% vs. 20% (*p* = 0.063, HR 0.52; 95% CI- 0.97–2.37)	HF hospitalization (HR0 1.79; 95% CI- 1.08–2.95; *p* = 0.022) and outpatient visits (250 vs. 84, *p* < 0.0001) were higher in the monitored group
2011	COMPASS-HF [89]	274 HF patients, on OMT, NYHA III-IV and ADHF hospitalization in previous 6 months	Implantable RV and ePAD pressure monitor	6 months	Nonsignificant 21% reduction in HF hospitalizations (*p* = 0.33)	time to first HF-related hospitalizations was 35% lower (HR-0.64, 95% CI-0.42–0.96, *p* = 0.03)
2016	CHAMPION [92]	550 HF patients with previous ADHF hospitalization and NYHA III	Implantable PA pressure monitor	18 months (complete follow up)	ADHF admissions were 33% lower (HR- 0.67, 95% CI 0.55–0.80, *p* < 0.0001)	

Acute decompensated heart failure (ADHF), New-York Heart Association functional class (NYHA), Hazard ratio (HR), Confidence interval (CI), Left ventricular ejection fraction (LVEF), Blood pressure (BP), Electrocardiograph (ECG), Cardiac implanted electronic devices (CIED), Optimal medical treatment (OMT), Heart failure (HF), Right ventricle (RV), Estimated pulmonary artery diastolic (ePAD).
